# Circulating and PBMC Lp-PLA_2_ Associate Differently with Oxidative Stress and Subclinical Inflammation in Nonobese Women (Menopausal Status)

**DOI:** 10.1371/journal.pone.0029675

**Published:** 2012-02-16

**Authors:** Jean Kyung Paik, Ji Young Kim, Oh Yoen Kim, Yonghee Lee, Tae-Sook Jeong, Gary Sweeney, Yangsoo Jang, Jong Ho Lee

**Affiliations:** 1 Yonsei University Research Institute of Science for Aging, Yonsei University, Seoul, Korea; 2 National Research Laboratory of Clinical Nutrigenetics/Nutrigenomics, Department of Food and Nutrition, College of Human Ecology, Yonsei University, Seoul, Korea; 3 National Research Laboratory of Lipid Metabolism and Atherosclerosis, Korea Research Institute of Bioscience and Biotechnology, Daejeon, Korea; 4 Institut Pasteur Korea, Seoul, Korea & Department of Biology, York University, Toronto, Canada; 5 Cardiology Division, Yonsei University College of Medicine, Seoul, Korea; 6 Cardiovascular Genome Center, Yonsei University College of Medicine, Seoul, Korea; 7 Severance Medical Research Institute, Yonsei University College of Medicine, Seoul, Korea; I2MC INSERM UMR U1048, France

## Abstract

**Background:**

This study aimed to determine the association of lipoprotein-associated phospholipase A_2_ (Lp-PLA_2_) activity in circulation and peripheral blood mononuclear cells (PBMCs) with inflammatory and oxidative stress markers in nonobese women and according to menopausal status. Lp-PLA_2_ activity, a marker for cardiovascular risk is associated with inflammation and oxidative stress.

**Methodology/Principal Findings:**

Eighty postmenopausal women (53.0±4.05 yr) and 96 premenopausal women (39.7±9.25 yr) participated in this study. Lp-PLA_2_ activities, interleukin (IL)-6, tumor necrosis factor (TNF)-α, and IL-1β in plasma as well as in PBMCs were measured. Plasma ox-LDL was also measured. Postmenopausal women demonstrated higher circulating levels of ox-LDL and IL-6, as well as IL-6, TNF-α, and IL-1β in PBMCs, than premenopausal women. In both groups, plasma Lp-PLA_2_ activity positively correlated with Lp-PLA_2_ activity in PBMCs and plasma ox-LDL. In premenopausal women, Lp-PLA_2_ activities in plasma and PBMCs positively correlated with IL-6, TNF-α, and IL-1β in PBMCs. In postmenopausal women, plasma ox-LDL positively correlated with PBMC cytokine production. In subgroup analysis of postmenopausal women according to plasma ox-LDL level (median level: 48.715 U/L), a significant increase in Lp-PLA_2_ activity in the plasma but not the PBMCs was found in the high ox-LDL subgroup. Plasma Lp-PLA_2_ activity positively correlated with unstimulated PBMC Lp-PLA_2_ activity in the low ox-LDL subgroup (r = 0.627, P<0.001), whereas in the high ox-LDL circulating Lp-PLA_2_ activity positively correlated with plasma ox-LDL (r = 0.390, P = 0.014) but not with Lp-PLA_2_ activity in PBMCs.

**Conclusions/Significance:**

The lack of relation between circulating Lp-PLA_2_ activity and Lp-PLA_2_ activity in PBMCs was found in postmenopausal women with high ox-LDL. This may indicate other sources of circulating Lp-PLA_2_ activity except PBMC in postmenopausal women with high ox-LDL. We also demonstrated that circulating Lp-PLA_2_ and PBMC secreted Lp-PLA_2_ associate differently with markers of oxidative stress and sub clinical inflammation in nonobese women, particularly according to the menopausal states.

## Introduction

Lipoprotein-associated phospholipase A_2_ (Lp-PLA_2_), also known as plasma platelet activating factor acetylhydrolase (PAF-AH), is unique among members of the phospholipase A_2_ superfamily due to its origin, its association with circulating lipoproteins, and its substrate preference for polar phospholipids, including those generated during the oxidation of low-density lipoprotein (LDL) [1]. Lp-PLA_2_ is secreted by monocytes, macrophages, T lymphocytes, and mast cells [2], and catalyzes the hydrolysis of oxidized LDL (ox-LDL) [3], which produces the proinflammatory mediators lysophosphatidylcholine and oxidized fatty acid [3]. There has been a growing interest in Lp-PLA_2_ because of its key role in lipid metabolism and in initiating inflammation [4]. Epidemiological and clinical studies have indicated that Lp-PLA_2_ is a marker for cardiovascular risk, with higher plasma Lp-PLA_2_ mass or activity correlating with a higher risk for cardiovascular events independent of systemic inflammation and other conventional risk factors [5]–[11].

Many studies found correlations between Lp-PLA_2_ and triglycerides, LDL-cholesterol, high-density lipoprotein (HDL)-cholesterol, body mass index (BMI), age, sex, use of postmenopausal hormones, and smoking [6], [9], [12]–[19]. Lp-PLA_2_ has been associated with an increased incidence of ischemic stroke among nonusers of hormone therapy in postmenopausal women independent of traditional cardiovascular risk factors. Furthermore, Keyzer et al. [20] found a positive association between Lp-PLA_2_ activity and inflammation and oxidative stress in a hypercholesterolemic swine model for atherosclerosis. Wang et al. [21] reported the stimulatory effect of ox-LDL on the expression of Lp-PLA_2_ in monocytes, which are a primary source of this enzyme. These recent findings in animal and in vitro studies may provide insight into the interaction between Lp-PLA_2_ activity and oxidative stress in the context of atherosclerosis. Therefore, our aim was to study the relationship of Lp-PLA_2_ activity in plasma and the enzyme activity in supernatants from nonstimulated peripheral blood mononuclear cell (PBMC) cultures. Plasma ox-LDL and cytokine production from PBMCs in healthy nonobese women and also according to the menopausal status were evaluated.

## Methods

### Study participants

A total of 176 healthy, nonobese women aged 20–68 years were recruited during routine check-ups at a health promotion center at Yonsei University Hospital. Postmenopausal status (n = 80) was defined as an absence of menstruation for at least 12 months and the presence of estrogen deficiency symptoms, including hot flushes, increased sweating, nervousness, irritability, depression, palpitations, insomnia, headaches, dyspareunia, and joint pains. Premenopausal status (n = 96) was defined as the presence of regular menses. At the time of subject enrollment, subjects were interviewed about smoking status (non-/ex-smoker and current smoker), and frequency of alcohol intake. Alcohol consumption was calculated as the grams of ethanol ingested per day and cigarettes smoking data were reported as the number of cigarettes smoked per day. All participants were clinically healthy and were not taking any medications known to affect the immune system, such as oral contraceptives, lipid-lowering agents, anti-hypertensive drugs, functional foods, or vitamin and/or mineral supplements. The purpose of the study was carefully explained to all participants and their written consent was obtained prior to their participation. The study design was approved by the Institutional Review Board of Yonsei University.

### Anthropometric parameters, blood pressure, and blood collection

Body weight and height were measured in the morning, lightly clothed without shoes and the BMI was calculated as body weight in kilograms divided by height in meters squared. Waist circumference was measured at the umbilical level with the subjects standing after normal expiration and the hip girth was measured at the widest part of the hip and, the waist and hip ratio (WHR) was calculated.

Blood pressure (BP) was measured in the left arm of seated patients with an automatic blood pressure monitor (TM-2654, A&D, Tokyo, Japan) after a 20-min rest. After a 12-hour fast, venous blood specimens were collected in EDTA-treated or untreated tubes. The blood specimens collected in the EDTA-treated tubes were used for the isolation of PBMCs or separated into plasma and stored at −70°C until further analysis. The blood samples collected in non-treated tubes were separated into serum and stored until further analysis.

### Serum lipid profile, fasting glucose, free fatty acid, and white blood cell count

Fasting total-cholesterol and triglyceride levels were measured using commercially available kits on a Hitachi 7150 Autoanalyzer (Hitachi Ltd., Tokyo, Japan). After precipitation of serum chylomicrons with dextran sulfate magnesium, the concentrations of LDL- and HDL-cholesterol in the supernatants were enzymatically measured. Fasting glucose levels were measured using a glucose oxidase method with a Beckman Glucose Analyzer (Beckman Instruments, Irvine, CA, USA). Free fatty acids were analyzed with a Hitachi 7150 autoanalyzer (Hitachi Ltd, Tokyo, Japan).White blood cell (WBC) count was determined using the HORIBA ABX diagnostic (HORIBA ABX SAS, Parc Euromedicine, France).

### Cytokine secretion in non-stimulated PBMCs

Whole blood was mixed with the same volume of RPMI 1640 (Gibco, Invitrogen, Carlsbad, CA, USA) and gently laid on a histopaque-1077 (Sigma-Aldrich, St. Louis, MO, USA). The sample was then centrifuged at 2000 rpm for 20 min at 10°C. After the separation, a thin layer of PBMCs was isolated and washed twice with RPMI 1640. The pellet was resuspended in RPMI 1640 with streptomycin. Isolated PBMCs were cultured in RPMI 1640 supplemented with 10% fetal bovine serum, seeded in 12-well plates (1×10^6^ cells/mL; Nunc, Roskilde, Denmark), and incubated at 37°C with 5% CO_2_ for 24 hours. After a 24-hour incubation, supernatants were collected and stored at −80°C until interleukin (IL)-1β, IL-6, tumor necrosis factor (TNF)-α, and Lp-PLA_2_ activity levels were assayed [22], [23].

### Cytokine assay for IL-1β, IL-6, and TNF-α levels in serum and PBMC supernatants

Levels of IL-1β, IL-6, and TNF-α in serum and PBMC supernatants were measured using the Bio-Plex™ Reagent Kit on the Bio-Plex™ (Bio-Rad Laboratories, Hercules, CA, USA), according to the manufacturer's instructions.

### Lp-PLA_2_ activity in plasma and PBMC supernatants

Lp-PLA_2_ activity in plasma and PBMC supernatants was measured by using a modification of a previously described high-throughput radiometric activity assay [11].

### Plasma-oxidized LDL and serum high sensitivity-C-reactive protein (hs-CRP)

Plasma ox-LDL was measured using an enzyme immunoassay (Mercodia, Uppsala, Sweden). The resulting color reaction was read at 450 nm with a Wallac Victor^2^ multilabel counter (Perkin Elmer Life Sciences, Turku, Finland). Serum hs-c-reactive protein (CRP) levels were measured with an Express Plus™ auto-analyzer (Chiron Diagnostics Co., Walpole, MA, USA) using a commercially available, high-sensitivity CRP-Latex(II) ×2 kit (Seiken Laboratories Ltd., Tokyo, Japan).

### Urinary 8-epi-prostaglandin F_2α_ (8-epi-PGF_2α_) levels

Urine was collected in polyethylene bottles containing 1% butylated hydroxytoluene after a 12-hour fast. The bottles were immediately covered with aluminum foil and stored at −70°C until further analysis. The compound 8-epi-PGF_2α_ was measured using an enzyme immunoassay (BIOXYTECH urinary 8-epi-PGF_2α_™ Assay kit, OXIS International Inc., Portland, OR, USA). Urinary creatinine levels were determined using the alkaline picrate (Jaffe) reaction. Urinary 8-epi-PGF_2α_ levels are expressed as pmol/mmol creatinine.

### Serum follicle stimulating hormone (FSH) and 17ß-estradiol levels

Serum levels of FSH and 17ß-estradiol were measured using commercially-available kits (ADIVIA Centaur FSH and ADIVIA Centaur Estradiol, respectively, Siemens, USA) on the ADIVIA Centaur (ADIVIA Centur, Siemens).

### Data analysis

Statistical analyses were performed using SPSS version 12.0 for Windows (SPSS Inc., Chicago, IL, USA). The independent t-test was used to compare parameters between the two groups. One-way analysis of variance (ANOVA) with Bonferroni correction was used to test whether there were effects from menopausal state and plasma ox-LDL levels (below or above the median level) in postmenopausal women. General linear model (GLM) analysis was also performed with adjustment for age or BMI and alcohol consumption. Frequency was tested with the chi-square test. Pearson and partial correlation coefficients were used to examine relationships between variables. For descriptive purposes, mean values are presented using untransformed values. Results are expressed as the mean ± standard deviation (SD). A two-tailed value of P<0.05 was considered statistically significant.

## Results

### Clinical characteristics of study participants

In this study, postmenopausal women had a significantly higher BMI and included a lower percentage of alcohol drinkers (Table 1). Before and after adjusting for age, BMI and percentage of alcohol drinkers, postmenopausal women demonstrated significantly higher WHRs in addition to higher serum levels of total-cholesterol, LDL-cholesterol, and glucose, and lower serum levels of hs-CRP, than premenopausal women. Postmenopausal women also had significantly lower serum levels of 17β-estradiol and higher FSH levels than premenopausal women. These differences confirmed that postmenopausal women had estrogen deficiency. Premenopausal and postmenopausal women did not differ in terms of diastolic BP, or serum levels of triglycerides, HDL-cholesterol, and free fatty acid. Premenopausal and postmenopausal women also did not differ in terms of the number of circulating leukocytes (Table 1).

**Table 1:** Clinical characteristics of the study participants according to menopausal status.

	Premenopausal women (n = 96)			Postmenopausal women (n = 80)			P_0_	P_1_
Age (yr)	39.7	±	9.25	53.0	±	4.05	<0.001	-
Years since menopause		-		3.49	±	3.87	-	-
Body Mass Index (kg/m^2^)	21.9	±	2.84	22.8	±	2.27	0.024	-
Cigarette smoker, n (%)	1 (1.0)			2 (2.5)			0.592	-
Alcohol drinker, n (%)	62 (64.6)			37 (46.3)			0.022	-
Waist hip ratio	0.84	±	0.05	0.88	±	0.05	<0.001	0.042
Systolic BP (mmHg)	109.3	±	14.2	118.6	±	12.0	<0.001	0.614
Diastolic BP (mmHg)	74.2	±	10.6	76.0	±	8.75	0.238	0.592
Triglyceride (mg/dL)∮	90.7	±	41.6	98.2	±	45.4	0.489	0.340
Total-cholesterol (mg/dL)	182.6	±	26.4	210.3	±	32.7	<0.001	0.004
LDL-cholesterol (mg/dL)	109.1	±	23.4	132.5	±	28.6	<0.001	0.006
HDL-cholesterol (mg/dL)	55.4	±	12.8	58.1	±	14.1	0.185	0.145
Glucose (mg/dL)∮	85.5	±	7.98	92.1	±	12.3	<0.001	0.016
Free fatty acid (uEq/L)∮	407.1	±	191.8	409.5	±	158.2	0.488	0.548
hs-CRP (mg/dL)∮	0.51	±	0.75	0.36	±	0.92	<0.001	<0.001
Serum FSH (IU/L)	10.6	±	17.9	75.2	±	28.9	<0.001	<0.001
Serum 17ß-estradiol (pg/mL)∮	134.6	±	126.9	18.2	±	24.8	<0.001	<0.001
White blood cells (×10^9^/L)∮	4.94	±	1.01	4.98	±	1.15	0.785	0.948

Means ± SD. Tested by independent t-test or general linear model with the adjustment.

∮ tested by log-transformed P_0_: unadjusted, P_1_: adjusted for age, BMI, and alcohol consumption.

### Oxidative stress markers, cytokines, and Lp-PLA_2_ activity according to menopausal status

Table 2 provides data for oxidative stress markers, circulating levels of cytokines, and Lp-PLA_2_ activity in the plasma and in supernatants from non-stimulated PBMC cultures from premenopausal and postmenopausal women. Significant differences between the two groups are indicated in the last column before and after adjustment for age, BMI and percentage of alcohol drinkers. Plasma ox-LDL and serum IL-6 levels in the postmenopausal group were significantly higher than those in premenopausal women. Urinary levels of 8-epi-PGF_2α_, serum levels of TNF-α and IL-1β, and Lp-PLA_2_ activities in plasma and cultured nonstimulated PBMC supernatants did not significantly differ between the two groups. Additionally, plasma Lp-PLA_2_ activity was not significantly difference between the two groups after further adjustment for LDL-cholesterol (p = 0.746). Nonstimulated PBMCs from postmenopausal women secreted significantly higher amounts of IL-6, TNF-α, and IL-1β into the culture media than those from premenopausal women (Table 2).

**Table 2:** Oxidative stress markers, cytokines, and Lp-PLA_2_ activity according to menopausal status.

	Premenopausal women (n = 96)			Postmenopausal women (n = 80)			P_0_	P_1_
Plasma oxidized LDL(U/L)∮	40.9	±	11.4	56.7	±	23.8	<0.001	0.031
Urinary 8-epi-PGF_2α_ (pg/mg creatinine)∮	1079.1	±	281.6	1199.8	±	461.5	0.179	0.400
Serum IL-6 (pg/mL)∮	1.94	±	1.28	4.53	±	5.27	<0.001	0.005
Serum TNF-α (pg/mL)∮	5.88	±	7.03	6.64	±	10.6	0.795	0.213
Serum IL-1ß (pg/mL)∮	0.70	±	1.17	0.77	±	1.53	0.929	0.961
Lp-PLA_2_ activity (nmol/mL/min)	28.3	±	9.42	30.6	±	7.77	0.084	0.447
Nonstimulated PBMC								
IL-6 (pg/mL)∮	786.1	±	3107.9	1409.0	±	6504.8	<0.001	0.027
TNF-α (pg/mL)∮	169.3	±	803.4	626.9	±	2044.3	<0.001	0.008
IL-1ß (pg/mL)∮	14.4	±	42.4	119.7	±	351.2	<0.001	<0.001
Lp-PLA_2_ activity (nmol/mL/min)	2.01	±	0.55	2.11	±	0.61	0.265	0.621

Mean ± SD. Tested by independent t-test or general linear model with the adjustment.

∮ tested by log-transformed. P_0_: unadjusted, P_1_: adjusted for age, BMI and alcohol consumption.

### Correlations among oxidative stress markers, cytokines, and Lp-PLA_2_ activity in the circulation and in PBMCs according to menopausal status

In both premenopausal and postmenopausal women, plasma Lp-PLA_2_ activity positively correlated with plasma ox-LDL and supernatant Lp-PLA_2_ activity from nonstimulated PBMC cultures (Table 3). In premenopausal women, Lp-PLA_2_ activity in plasma and PBMC supernatants positively correlated with IL-6, TNF-α, and IL-1β levels in nonstimulated PBMCs. After the adjustment for age, BMI, alcohol consumption, the relationships were still retained (IL-6 and IL-1β). In postmenopausal women, plasma ox-LDL positively correlated with IL-6, TNF-α, and IL-1β levels in cultured nonstimulated PBMC supernatants. After the adjustment, the relationships were still retained (TNF-α and IL-1β) (Table 3). IL-6, TNF-α, and IL-1β levels in nonstimulated PBMCs were linearly interrelated for both premenopausal and postmenopausal women (data not shown). In the postmenopausal women, serum IL-6 levels positively correlated with serum IL-1β (r = 0.396, P<0.001) and serum TNF-α (r = 0.296, P = 0.012). Serum IL-6 levels negatively correlated with PBMC levels of IL-6 (r = −0.299, P = 0.005), TNF-α (r = −0.359, P = 0.001), and IL-1β (r = −0.302, P = 0.005) in premenopausal women, whereas serum IL-6 levels positively correlated with PBMC levels of IL-6 (r = 0.317, P = 0.006), TNF-α (r = 0.313, P = 0.007), and IL-1β (r = 0.316, P = 0.006) in postmenopausal women.

**Table 3:** Correlations among oxidative stress markers, cytokines, and Lp-PLA_2_ activity in the circulation and in PBMCs according to menopausal status.

	Premenopausal women (n = 96)						Postmenopausal women (n = 80)					
	Plasmaox-LDL		Plasma Lp-PLA_2_ activity		Nonstimulated PBMC Lp-PLA_2_		Plasma ox-LDL		Plasma Lp-PLA_2_ activity		Nonstimulated PBMC Lp-PLA_2_	
	r_0_	r_1_	r_0_	r_1_	r_0_	r_1_	r_0_	r_1_	r_0_	r_1_	r_0_	r_1_
Plasma ox- LDL(U/L)∮	-	-	0.244*	0.247*	0.022	0.006	-	-	0.390***	0.394**	0.215	0.154
Serum IL-6 (pg/mL)∮	0.042	0.051	−0.150	−0.154	−0.020	−0.032	0.171	0.143	0.022	0.016	0.019	−0.017
Serum TNF-α (pg/mL)∮	−0.037	−0.016	−0.178	−0.202	0.101	0.068	0.090	0.031	−0.022	−0.018	0.030	−0.008
Serum IL-1ß (pg/mL)∮	−0.018	−0.020	0.042	0.033	0.073	0.042	−0.061	−0.062	−0.050	−0.046	−0.022	−0.048
Lp-PLA_2_ activity (nmol/mL/min)	0.244*	0.247*	-	-	0.427***	0.422***	0.390***	0.394**	-	-	0.380**	0.374**
Nonstimulated PBMCs												
IL-6 (pg/mL)∮	0.058	0.042	0.326**	0.318**	0.363***	0.353**	0.230*	0.222	0.051	0.073	0.127	0.121
TNF-α (pg/mL)∮	0.106	0.072	0.209*	0.202	0.205*	0.198	0.308**	0.307**	0.171	0.208	0.032	0.022
IL-1ß (pg/mL)∮	0.056	0.035	0.277**	0.269**	0.272**	0.255*	0.304**	0.301**	0.220	0.251*	0.080	0.067
Lp-PLA_2_ activity (nmol/mL/min)	0.022	0.006	0.427***	0.422***	-	-	0.215	0.154	0.380**	0.374**	-	-

*Pearson* and *partial* correlation analysis, r_0_: unadjusted, r_1_: adjusted for age, BMI, and alcohol consumption,

*P<0.05,

**P<0.01,

***P<0.001,

∮ tested by log-transformed.

### Lp-PLA_2_ activity, oxidative stress markers, and LDL-cholesterol according to menopausal status and plasma ox-LDL levels

**Figure 1:**
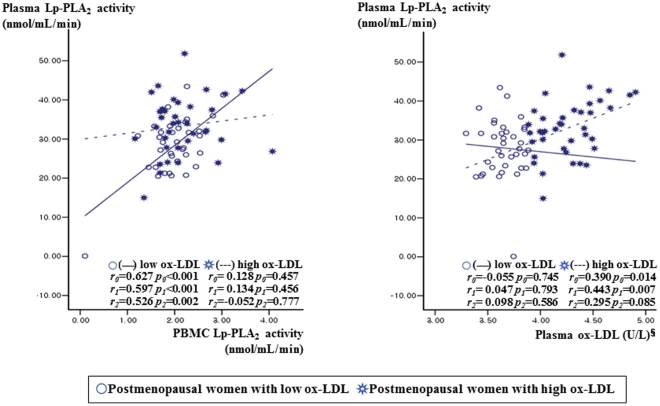
Relationship between Lp-PLA_2_ activity from PBMCs or plasma ox-LDL and plasma Lp-PLA_2_ activity according to plasma ox-LDL (below or above the median level of 48.715 U/L) in postmenopausal women. ^§^tested by log-transformed. Tested by Pearson correlation (*r_0_*) or partial correlation analysis (*r_1_, r_2_*). *r*_0_: correlation coefficient, unadjusted. *r_1_*: correlation coefficient after adjusted for age, BMI, and alcohol consumption. *r_2_*: correlation coefficient after adjusted for age, BMI, alcohol consumption, and LDL-cholesterol.

**Figure 2:**
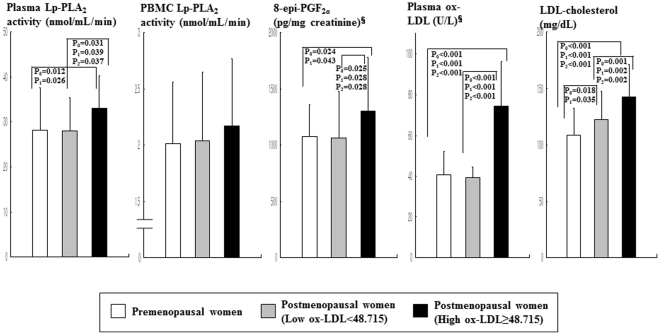
Lp-PLA_2_ activity in plasma and supernatants from nonstimulated PBMC cultures and oxidative stress markers according to menopausal status and plasma ox-LDL levels (below or above the median level of 48.715 U/L). Data are means ± SD. ^§^tested by log-transformed. P_0_: unadjusted, tested by one-way ANOVA with Bonferroni method P_1_: adjusted for BMI and alcohol consumption, tested by general linear model (GLM) analysis. P_2_: adjusted for age, BMI, and alcohol consumption, tested by GLM analysis.

Since Lp-PLA_2_ is known to hydrolyze the sn2 ester bond of oxidized phospholipids including ox-LDL [12], the postmenopausal women were subdivided into two groups according to plasma ox-LDL level: high ox-LDL (≥48.715 U/L, n = 40) and low ox-LDL (<48.715 U/L, n = 40) according to the median level of ox-LDL. In the postmenopausal women with low ox-LDL, plasma Lp-PLA_2_ activity positively correlated with unstimulated PBMC Lp-PLA_2_ activity (r_0_ = 0.627, P_0_<0.001; r_1_ = 0.597, P_1_<0.001) before and after the adjustment for age, BMI, and alcohol consumption (Fig. 1). In postmenopausal women with high ox-LDL, however, circulating Lp-PLA_2_ activity positively correlated with plasma ox-LDL (r_0_ = 0.390, P_0_ = 0.014; r_1_ = 0.443, P_1_ = 0.007) but not with Lp-PLA_2_ activity in PBMCs. Additionally partial correlation coefficient with adjust for age, BMI, alcohol consumption, and LDL-cholesterol level were also used to examine the relationships between variables. In the postmenopausal women with low ox-LDL, plasma Lp-PLA_2_ activity positively correlated with non-stimulated PBMC Lp-PLA_2_ activity (r_2_ = 0.526, P_2_ = 0.002) but not correlated with plasma ox-LDL (r_2_ = 0.098, P_2_ = 0.586). In postmenopausal women with high ox-LDL, the positive correlation between circulating Lp-PLA_2_ activity and plasma ox-LDL (r_2_ = 0.295, P_2_ = 0.085) was a little bit attenuated, but is in a positive direction. On the other hand, circulating Lp-PLA_2_ did not correlation with Lp-PLA_2_ activity in PBMCs (r_2_ = −0.052, P_2_ = 0.777). The postmenopausal group with high ox-LDL had higher levels of plasma Lp-PLA_2_ activity, urinary 8-epi-PGF_2α_ excretion, and serum LDL-cholesterol than the postmenopausal group with low ox-LDL, or the premenopausal group, before and after adjustment for age or BMI and percentage of alcohol drinkers (Fig. 2). In addition, the postmenopausal group with low ox-LDL had higher serum levels of LDL-cholesterol than the premenopausal group.

## Discussion

The major finding of this study is the lack of relation between circulating Lp-PLA_2_ activity and Lp-PLA_2_ activity in PBMCs in postmenopausal women with high ox-LDL (≥48.715 U/L, above median). A significant increase in Lp-PLA_2_ activity in the plasma but not the PBMCs of postmenopausal women with high ox-LDL may indicate other sources of Lp-PLA_2_ production except PBMC. The extent of the increase in plasma Lp-PLA_2_ may depend not only on the levels of lipoproteins carrying Lp-PLA_2_ in circulation but also on the cellular synthesis of this enzyme [24]. Monocytes, macrophages, T-lymphocytes, mast cells, and liver cells are known as the main sources of Lp-PLA_2_ [25], [26]. Recently, Keyzer et al. [20] found increased circulating Lp-PLA_2_ activity with increased ox-LDL levels in hypercholesterolemic pigs and the main source of increased circulating Lp-PLA_2_ activity were plaque macrophages [20], [27], [28]. Therefore, the Lp-PLA_2_ production in plaque macrophages could partly explain the positive correlation of circulating Lp-PLA_2_ activity with plasma ox-LDL but not with Lp-PLA_2_ activity in PBMCs from postmenopausal women with high ox-LDL in this study. However, we could not measure Lp-PLA_2_ activity in plaque macrophages, or the plaque or intima itself, where it may be of most biological relevance.

Lp-PLA_2_ is thought to play an atherogenic role by hydrolyzing oxidized phospholipids in ox-LDL, resulting in the generation of two bioactive lipid mediators, lysophosphatidyl choline, and oxidized free fatty acids [3], [24], [29]. The biological role of Lp-PLA_2_ is also controversial; initial reports indicated an antiatherogenic effect, whereas growing evidence has demonstrated a role for Lp-PLA_2_ as a proinflammatory molecule and an independent risk factor for CVD [5], [11], [14], [30], [31]. Lp-PLA_2_ belongs to the expanding superfamily of structurally diverse phospholipase A_2_ enzymes, also known as PAF-AH [32]. It travels mainly with LDL in the blood, and less than 20% is associated with HDL [1]. In mice, the majority of plasma PAF-AH is bound to HDL, this was considered as possible antiatherogenic effect [33]. In human atherosclerotic lesions, two main sources of Lp-PLA_2_ were identified in human atherosclerotic lesions, including that which is brought into the intima bound to LDL from the circulation, and that which is synthesized de novo by plaque inflammatory cells (i.e. macrophages, T cells, mast cells) [1], [32], [34]. Lp-PLA_2_ hydrolyzes the sn-2 ester bond of oxidized phospholipids, generating bioactive oxidized free fatty acids and lysophosphatidyl choline, which are potent proinflammatory and proatherogenic products [1]. Indeed, Stafforini et al. [35] showed that the secreted form of Lp-PLA_2_ released F_2_-isoprostanes, the end-products of lipid oxidation, from the sn-2 position of phosphatidylcholine with high affinity. Kono et al. [36] reported that intracellular type II Lp-PLA_2_, which shares homology with the plasma enzyme Lp-PLA_2_, is involved in the metabolism of esterified 8-iso-PGF_2α_. We also showed that mean levels of plasma Lp-PLA_2_ activity and urinary 8-epi-PGF_2α_ were higher among postmenopausal women with high ox-LDL than those with low ox-LDL or in premenopausal women.

Ox-LDL stimulates Lp-PLA_2_ expression in monocytes through the pathway of phosphatidylinositol 3-kinase and p38 mitogen-activating protein kinase [21], which mediates the expression of many genes involved in stress-induced responses (e.g., IL-1β) [37]. Shi et al. [24] found that the activation of leukocytes in the experimental model of diabetes and hypercholesterolemia is associated with a rapid increase in circulating Lp-PLA_2_ and an upregulation in a range of inflammatory mediators, including IL-6, TNF-α, and IL-1β. In the postmenopausal women in this study, plasma ox-LDL also positively correlated with IL-6, TNF-α, and IL-1β levels in cultured, nonstimulated PBMC supernatants. This observation supports the previous finding that the presence of ox-LDL significantly induced the expression of IL-1β, IL-6, and TNF-α, whereas unmodified LDL had no effect on the expression of inflammatory mediators [24]. This correlation, however, was not found in premenopausal women. Additionally, the postmenopausal women showed higher levels of plasma ox-LDL and serum IL-6, and more cytokine production from PBMCs than the premenopausal women. This result may be partly related to the exaggerated production of cytokines caused by estrogen deprivation after menopause. Rachon et al. [22] showed that estrogen deprivation after menopause enhanced IL-6 production by PBMCs and increased serum IL-6 levels in postmenopausal women. It has been also suggested that the increased production of cytokines in healthy, older individuals results from the loss of sex steroids [38]. Estradiol acts to inhibit pro-inflammatory cytokine gene expression, NF-κB binding, and production of proinflammatory cytokines [39], [40]. In addition, the levels of IL-6, TNF-α, and IL-1β in supernatants from nonstimulated PBMC cultures of the postmenopausal women positively correlated with serum IL-6 levels [41]. This finding supports the previous suggestion that locally-produced TNF-α and IL-1β do not escape into the circulation, although they induce a strong systemic IL-6 response [41]. Therefore, enhanced cytokine production by PBMCs in postmenopausal women, may not only result from the activation of leukocytes in the presence of high ox-LDL after menopause but also may be the consequence of the decrease in estrogen levels or other factors that contribute to the aging process [12], [22], [39]. On the other hand, cytokine production particularly IL-6 and IL-1β from PBMCs in premenopausal women, but not in postmenopausal women positively correlated with Lp-PLA_2_ activity in plasma and PBMC supernatants. The biological role of Lp-PLA_2_ or the activity levels according to the menopausal states are still controversial, but our results may be partly explained by the recent studies demonstrating that Lp-PLA_2_ works as an proinflammatory molecules or initiate inflammatory response, and is an independent risk factor for CVD [5], [11], [14], [30], [31]. It is known that circulating Lp-PLA_2_ activity is derived from atherosclerotic plaque cells, however our study subjects had not carried cardiovascular disease including atherosclerosis or other chronic diseases (i.e. diabetes, or dyslipidemia). Therefore, we could observe the clear positive correlation in premenopausal women rather than postmenopausal women whose metabolisms are interfered by the stressful condition (i.e. oxidative stress, estrogen deprivation).

In addition, the prospective observation needs to be performed in the future to investigate if Lp-PLA_2_ is a physiological responder or an inducer of vascular inflammation. Actually, the Lp-PLA_2_ activity may be different according to the ethnicities, for example, rare homozygous mutation of Lp-PLA_2_ V279F polymorphism, the F/F genotype indicating the loss of function of Lp-PLA_2_ activity and the less atherogenic properties is found in Korean but not in Western people [42]. Our study was designed for the cross-sectional observation, not for prospective observation, thus it is not easy to determine the casual relationship between Lp-PLA_2_ and inflammatory response. In summary, the lack of relationship between circulating Lp-PLA_2_ activity and Lp-PLA_2_ activity in PBMCs was found in postmenopausal women with high ox-LDL. This may indicate other sources of circulating Lp-PLA_2_ activity except PBMC in postmenopausal women with high ox-LDL. We also demonstrated that circulating Lp-PLA_2_ and PBMC secreted Lp-PLA_2_ associate differently with markers of oxidative stress and sub clinical inflammation in nonobese women, particularly according to the menopausal states.
